# The role of MHC class I gene products in SIV infection of macaques

**DOI:** 10.1007/s00251-017-0997-3

**Published:** 2017-07-10

**Authors:** Zachary A. Silver, David I. Watkins

**Affiliations:** 1 Medical Scientist Training Program, University of Miami Miller School of Medicine, Miami, FL USA; 2 Department of Pathology, University of Miami Miller School of Medicine, Miami, FL USA

**Keywords:** Major histocompatibility complex (MHC), Simian immunodeficiency virus (SIV), Human immunodeficiency virus (HIV), Cytotoxic T lymphocyte (CTL), Nonhuman primate (NHP) model for AIDS

## Abstract

Human immunodeficiency virus (HIV) remains among the most significant public health threats worldwide. Despite three decades of research following the discovery of HIV, a preventive vaccine remains elusive. The study of HIV elite controllers has been crucial to elaborate the genetic and immunologic determinants that underlie control of HIV replication. Coordinated studies of elite control in humans have, however, been limited by variability among infecting viral strains, host genotype, and the uncertainty of the timing and route of infection. In this review, we discuss the role of nonhuman primate (NHP) models for the elucidation of the immunologic correlates that underlie control of AIDS virus replication. We discuss the importance of major histocompatibility complex class I (MHC-I) alleles in activating CD8+ T-cell populations that promote control of both HIV and simian immunodeficiency virus (SIV) replication. Provocatively, we make the argument that T-cell subsets recognizing the HIV/SIV viral infectivity factor (Vif) protein may be crucial for control of viral replication. We hope that this review demonstrates how an in-depth understanding of the MHC-I gene products associated with elite control of HIV/SIV, and the epitopes that they present, can provide researchers with a glimpse into the protective immune responses that underlie AIDS nonprogression.

## Introduction

HIV remains among the most significant public health threats worldwide. In 2015, there were 1.1 million AIDS-related deaths and the number of people living with HIV exceeded 35 million (UNAIDS 2016). This year alone, the USA will spend tens of billions of dollars on efforts to fight HIV domestically and internationally. Although drug regimens have been developed to decrease transmission and morbidity, only half of HIV-infected individuals are aware of their infection status and less than half are receiving treatment. Further, access to anti-retroviral therapy (ART) is challenging in resource-poor settings, such as sub-Saharan Africa, where approximately two thirds of all HIV-positive individuals live and the vast majority of all new cases are diagnosed. More than 30 years into the epidemic, the need for an HIV vaccine has never been greater.

The major histocompatibility complex (MHC) is a highly polymorphic region of the vertebrate genome that plays a critical role in autoimmunity and host immune response to infection (Horton et al. 2004). The MHC, which lies on chromosome 6, has typically been divided into three regions: class I, class II, and class III. The class I gene products are expressed on virtually all nucleated cells and complex with β_2_ microglobulin to form a functional MHC class I (MHC-I) molecule. The MHC-I molecule is expressed on the cell membrane, where its highly variable peptide-binding domain presents peptides of approximately 9–11 amino acids in length. These peptides are normally derived from intracellular proteins that have been degraded by the proteasome. The presentation of pathogen-derived peptides by MHC-I molecules, however, provides a mechanism for CD8+ T-cells to survey and destroy cells that have been infected by viruses or parasites (Bontrop 2006).

## Factors involved in the development of HIV elite control

In the absence of treatment, HIV-1 infection progresses to AIDS in >99% of cases (Muenchhoff et al. 2016; Sabin and Lundgren 2013). Elite controllers represent a remarkable minority of individuals who maintain normal CD4+ T-cell counts and low or undetectable viral loads in the absence of ART (Gaardbo et al. 2012). The study of HIV elite controllers has provided in-depth knowledge of the viral, genetic, and immunologic correlates of HIV control. Such phenotypes have been instrumental in guiding our understanding of HIV biology and therapy. For example, the host genetic determinant *ccr5*Δ32 has been shown to facilitate elite control by modifying the HIV co-receptor on T lymphocytes in a way that precludes virus binding and entry to the cell. The discovery of the remarkable *ccr5*Δ32 phenotype led to the first and only case of a curative HIV treatment (Allers et al. 2011). The so-called Berlin patient has shown no signs of HIV infection since receiving a bone marrow transplant from a homozygous *ccr5*Δ32 donor (Hutter et al. 2009; Yukl et al. 2013).

In the early 1990s, Ronald C. Desrosiers’ laboratory obtained a blood sample from an elite controller in central Massachusetts and discovered that this patient’s virus remarkably displayed a deletion in the auxiliary gene *nef* (Kirchhoff et al. 1995; Mariani et al. 1996). Generation of an SIVmac239 mutant containing a similar deletion in *nef* and subsequent infection of rhesus macaques with this strain revealed a similar degree of attenuation and phenotype of infection in the nonhuman primate model (Kestler et al. 1991). The observation that deletions in HIV *nef* were associated with elite control led to a number of studies aimed at dissecting the functional role of Nef in the progression toward AIDS. In 1998, Kathleen Collins and colleagues demonstrated that CD8+ T-cells inefficiently lyse HIV-infected primary T lymphocytes (Collins et al. 1998). In contrast, lymphocytes infected with an HIVΔ*nef* strain could readily be lysed in the presence of cytotoxic T lymphocytes (CTLs). The group showed that cells infected with the wild-type HIV strain managed to escape recognition and lysis by CD8+ T-cells by decreasing the density of MHC-I, and its bound peptide, on the surface of the cell. In the absence of Nef, CTLs efficiently lysed HIV-infected target cells.

The evolutionary relationship between HIV Nef, CTL responses, and elite control is striking (Collins and Baltimore 1999). HIV Nef serves to downregulate MHC-I and limit CTL activation, and in the context of a *nef*-deficient virus, an individual does not progress to AIDS. The two experiments highlight the importance of MHC-I gene products for the activation of CTLs to control HIV infection. It is therefore not surprising that cohorts of elite controllers are often enriched with genetic variants that influence immunological outcomes, such as CD8+ T-cell recognition (*HLA*-B*27, B*57) and natural killer cell function (*KIR*-*3DS1*) (Alter et al. 2011; Martin and Carrington 2013; Mendoza et al. 2012; Wang et al. 2009). Indeed, although *HLA*-B*57 has a frequency of approximately 11% in the Caucasian US population, one study demonstrated that this allele was present in 11 of the study’s 13 elite controllers (85%) (Migueles 2000). In contrast, genotyping of the 200 HIV progressors in this study revealed an HLA-B*57 prevalence of only 9.5%. Taken together, these findings emphasize the relative importance of MHC-I gene products for the control of HIV replication.

## Importance of Vif-specific T-cells for spontaneous control of SIV replication

Development of a successful vaccine will require in-depth knowledge of the genetic and immunologic correlates underlying HIV control. To this end, researchers have investigated the immune responses of elite controllers and compared them to those of progressors to gain insight into the nature of protective immunity against HIV (Ahlers and Belyakov 2010). Coordinated studies of elite control in humans have, however, been limited by variability among infecting viral strains, host genotype, and the uncertainty of the timing and route of infection (Deymier et al. 2015; Loffredo et al. 2007a; Weintrob et al. 2003). It should not be surprising, therefore, that researchers have turned their attention to the study of nonhuman primate (NHP) models. Humans and rhesus macaques have similar immune systems, and SIV, the causative agent of AIDS in macaques, has a similar pathogenesis and sequence homology to HIV (Nathanson et al. 1999; Regier and Desrosiers 1990). Thus, tightly controlled experiments using rhesus macaques have become an essential tool to model control of the immunodeficiency virus in humans.

Studies in NHPs have suggested that allelic variation in the MHC-I genes and the CD8+ T-cells that bind to their gene products play a critical role in controlling SIV and HIV (Carrington and O’Brien 2003; Goulder and Watkins 2008). Our laboratory and others have demonstrated that depletion of CD8+ T-cells in elite controller rhesus macaques is associated with a rise in viral loads, which subsequently wanes as SIV-specific CD8+ T-cell subsets re-emerge (Friedrich et al. 2007). The presence of SIV-specific T-cells has also been associated with reduced peak viremia and a slower rate of disease progression (Borrow et al. 1994). Further, high frequencies of CD8+ T-cells can exert strong selective pressure on replicating viruses and cause viral escape and persistent infection (Goulder et al. 1997, 2001). Such escape mutants, however, can exact a toll on viral fitness and will revert in the absence of CD8+ T-cell pressure (Friedrich et al. 2004; Leslie et al. 2004). Given the importance of CD8+ T-cells in the control of viral replication, efforts have been made to characterize the MHC-I-restricted epitopes associated with CD8+ T-cell responses that control infection.

The characterization of rhesus macaque MHC-I alleles, their peptide-binding motifs, and the SIV epitopes that they present have helped elucidate the mechanisms involved in control of AIDS virus replication (Allen et al. 1998; Dzuris et al. 2000; Loffredo et al. 2004, 2005, 2009). Our laboratory and others have made a significant effort to map the SIV epitopes restricted by different MHC-I alleles and identify the epitopes associated with control of viremia. The immunodominance hierarchy of protective CD8+ T-cell responses, however, is complex and their efficacy depends on activation, potency, viral fitness, and an individual’s expressed MHC-I alleles. Knowledge of a macaque’s MHC-I alleles and the SIV peptides they present is crucial to elaborate the characteristics of effective CD8+ T-cell responses. The use of tetramers, ELISpot, and intracellular cytokine staining (ICS) has allowed researchers to describe the relative frequencies of SIV-specific T-cells and establish correlations between frequencies of T-cell subsets and SIV viral loads.

The mapping of MHC-I alleles and the epitopes they bind in the context of elite controller macaques has shown that CD8+ T-cells in these monkeys appear to focus on the SIV viral infectivity factor (Vif) protein. Epitopes from Vif are presented by numerous MHC-I alleles (Table 1), including those that are associated with spontaneous elite control of SIV (Mamu-B*17 and Mamu-B*08). In 2002, a study by Mothe et al. found that Mamu-B*17 binds 50 peptides from 7 different SIV proteins (Mothe et al. 2002). Only 16 of these peptides, however, proved capable of eliciting IFN-γ production by cytotoxic T lymphocytes in vitro. Of these 16 peptides, 5 were epitopes derived from Vif, which is surprising given the relatively small size of Vif (214 amino acids) compared to other SIV proteins. Half of all rhesus macaques expressing Mamu-B*08 will spontaneously become elite controllers after infection with SIVmac239 (Loffredo et al. 2007b). Loffredo et al. mapped the Mamu-B*08-restricted CD8+ T-cell responses to 13 epitopes across Gag, Vpr, Env, Vif, Nef, and Rev. The strongest and most frequent immune responses were against epitopes in Vif, Nef, and Rev, and sequence analysis of SIV quasispecies from these animals predominantly demonstrated mutations in Vif and Nef epitopes. In a set of follow-up experiments, we made clear the importance of Vif-specific T-cells by demonstrating that escape mutations in the Vif RL8 epitope differentiate Mamu-B*08+ progressors from elite controllers (Mudd et al. 2012a).

**Table 1:** The minimal optimal SIV Vif epitopes required for CD8+ T-cell recognition

MHC-I protein	Amino acid positions	Length	Short name	Amino acid sequence	Reference
Mamu-B*08	123–131	9	RL9	RRAIRGEQL	Loffredo et al. (2007b)
	172–179	8	RL8	RRDNRRGL	Loffredo et al. (2007b)
Mamu-B*17	44–52	9	HW9	HFKVGWAWW	Mothe et al. (2002)
	66–73	8	HW8	HLEVQGYW	Mothe et al. (2002)
	135–143	9	CY9	CRFPRAHKY	Mothe et al. (2002)
Mamu-A*01	100-109	10	VL10	VTPNYADILL	Sidney et al. (2000)
	144–152	9	QA9	QVPSLQYLA	Allen et al. (2001)
Mamu-A*02	89–97	9	IW9	ITWYSKNFW	Loffredo et al. (2004)
	97–104	8	WY8	WTDVTPNY	Loffredo et al. (2004)
	104–113	10	YY10	YADILLHSTY	Loffredo et al. (2004)
Mamu-A*07	145-153	9	VL9	VPSLQYLAL	Reed et al. (2011)

Amino acids from SIV Vif are presented across a wide-range of MHC-I molecules. CD8+ T-cell responses against these epitopes have been associated with IFN-γ production, control of viral replication, and/or selection of viral escape mutants.

The remainder of this review will focus on the nature of the antigen-specific T-cell responses that may be responsible for HIV and SIV control in the context of protective MHC-I alleles in humans and the NHP model of AIDS. Specifically, we will focus on Vif as a target for CD8+ T-cells that effectively control viral replication. We will first describe Vif’s function, protein targets, and relationship to MHC-I presentation of peptides. We will then present evidence that Vif-specific CD8+ T-cells are an important component of an effective immune response to SIV. Finally, we will describe studies that provide evidence suggesting that Vif-specific T-cell responses are an important component for the prevention and control of HIV infection in humans.

## An unusual relationship between HIV/SIV Vif, APOBEC3G, and MHC-I epitope presentation

In 1998, the Desrosiers Laboratory published a study showing that a *vif*-deficient mutant of SIVmac239 has decreased replicative capacity and is weakly infectious in rhesus macaques (Desrosiers et al. 1998). In line with these findings, several other groups made the observation that *vif*-deficient HIV-1 strains can only complete one round of replication. After that, the progeny virus is predominantly non-infectious (Chowdhury et al. 1996). The inability of *vif*-deficient HIV-1 strains to undergo productive replication triggered a widespread search for the host targets of Vif and ultimately led to the discovery of the viral restriction factor APOBEC3G (A3G) (Sheehy et al. 2002). A3G’s importance as a viral restriction factor quickly became clear, with studies demonstrating that knockdown of A3G in non-permissible cells could render them permissible to HIV-1 infection (Sadler et al. 2010).

A3G belongs to a family of proteins called activation-induced cytidine deaminases (Harris and Liddament 2004). In the case of lentiviruses, A3G and other family members target the single-stranded DNA generated by reverse transcription of the viral genome. Through removal of an amine group, a cytosine in the DNA negative strand is converted into a uracil, thereby leading to a G ➔ A mutation upon generation of the positive DNA stand. These mutations have major consequences for the viral genome, as 20% of all A3G-induced mutations result in stop codons. Stop codons can lead to an abundance of truncated proteins in the endoplasmic reticulum and thereby maintain a plentiful supply of peptides for MHC-I binding and the induction of CD8+ T-cell responses (Casartelli et al. 2010). A3G-induced mutations can also restrict HIV replication in other ways, for example, by interfering with HIV integration into the host genome and triggering host DNases to degrade the mutated viral DNA.

The role of HIV Vif, therefore, is to prevent the incorporation of A3G into viral particles by targeting it for proteasomal degradation. To achieve this, Vif hijacks the T-cell transcription factor CBF-β, which stabilizes Vif and promotes its binding to Elongin B/C and Cullin-5 to form the Cul5-RING ubiquitin ligase (Donahue et al. 2008; Yu et al. 2003). This E3 ubiquitin ligase complex, with Vif and A3G attached, recruits E2 ubiquitin conjugating enzyme, thus allowing for ubiquitination of A3G. By targeting A3G to the proteasome, the virus can prevent the inclusion of damaging amounts of A3G into its capsids and continue its lifecycle.

## Vif-specific T-cells are associated with vaccine-induced control of SIV replication

**Fig. 1:**
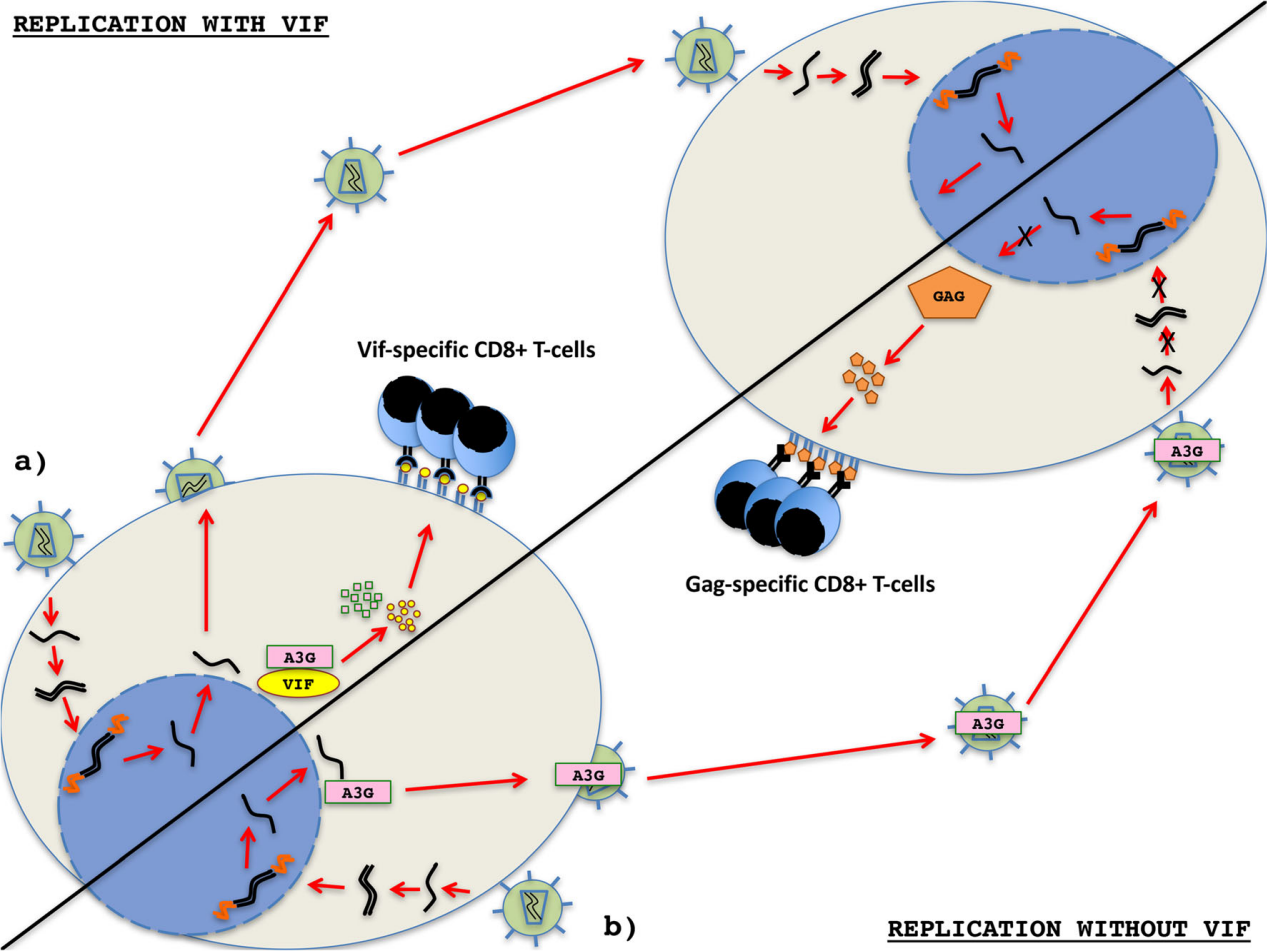
Relationship between APOBEC3G, Vif, MHC presentation, and CTL responses. **a** Proteasomal degradation of the Vif/A3G complex limits incorporation of A3G into progeny virions, thereby preventing hypermutation during subsequent cellular infection. This proteasomal degradation may lead to increased presentation of Vif epitopes in the context of MHC-I gene products and provide a target for Vif-specific CTLs. Vif-specific T-cell responses may therefore be key for control of HIV/SIV by targeting cells that evade the activity of the A3G restriction factor. **b** In the absence of Vif, A3G is incorporated into progeny virions. Upon infection of subsequent cells, A3G causes mutations during reverse transcription of viral RNA that result in replication-incompetent virus

The expression of Vif is clearly important for effective HIV replication. However, in targeting A3G to the proteasome, Vif may inadvertently be degraded and peptide sequences from the protein might be presented in the context of MHC-I gene products (Dang et al. 2008). Given the importance of Vif for enabling viral replication, it is tempting to speculate that the most important cellular reservoirs of HIV/SIV would consist of cells where Vif is either moderately or highly expressed. Although these cells would produce replication-competent HIV, the proteasomal degradation of Vif could ultimately lead to an increased presentation of Vif epitopes in the context of MHC-I proteins. This increased density of Vif epitopes on the surface of the cell could serve as a potent target for Vif-specific CTLs to control infection and limit the production of replication-competent virus (Fig. 1).

Given the relative abundance of Vif peptides that are bound and presented by MHC-I molecules, it is not surprising that many rhesus macaques develop Vif-specific CD8+ T-cell responses. Our laboratory and others have associated several of these unique Vif-specific T-cell subsets with control of SIV infection (Table 1). In 2007, we depleted CD8+ T-cells from elite controllers, which abruptly triggered a resurgence of plasma viral loads (Friedrich et al. 2007). The reemergence of CD8+ T-cells recognizing the Vif HW8 epitope, a Mamu-B*17-restricted epitope, coincided with a decrease in viral load and subsequent reestablishment of control. In another set of experiments, depletion of CD8+ T-cells in elite controllers led to expansion of CTLs recognizing the Mamu-B*08-restricted Vif RL8 epitope (Loffredo et al. 2007a). Interestingly, these CD8+ T-cell responses selected for mutations that facilitated escape from Vif RL8-specific CD8+ T-cells, demonstrating the considerable pressure that these CTLs exert on SIV replication.

Although single subsets of epitope-specific CTLs can potently kill SIV-infected cells, it has become clear that broad CD8+ T-cell responses are important for the control of SIV. Data from our laboratory demonstrated that a Mamu-B*08+ rhesus macaque with high viral loads in the chronic phase mounted 87% of their CTL responses against a single epitope, Vif RL8 (Loffredo et al. 2008). The controllers, on the other hand, showed high frequencies of RL8-specific CTLs (∼54%), but also subdominant frequencies of CTLs that recognized Vif RL9, Env, and Nef. In another experiment, Martins et al. demonstrated that broad T-cell responses after vaccination and heterologous challenge with SIVsmE660 are associated with markers of delayed disease progression (Martins et al. 2010). In particular, the number of different Vif epitopes recognized by CTLs in vaccinees was associated with reduced peak viremia and higher CD4+ T-cell counts during the chronic phase of infection. These data suggest that control of viral replication requires pressure from multiple CTL populations to maintain low viral loads, perhaps in order to minimize the chances of evolutionary escape. Therefore, increasing the breadth of vaccine-induced Vif-specific T-cell responses could provide an important means to control SIV replication.

The range of CTL breadth required for successful control of SIV infection is not completely clear. Our laboratory previously demonstrated that escape mutants in Mamu-B*08-restricted Vif epitopes differentiate elite controller macaques from progressors (Loffredo et al. 2007a; Mudd et al. 2012a). Given this intriguing data, Mudd et al. tested whether vaccination with three Mamu-B*08-restricted CD8+ T-cell epitopes—Vif RL8, Vif RL9, and Nef RL10—using a recombinant yellow fever 17D prime with a recombinant adenovirus serotype 5 boost was sufficient to induce control of SIV (Mudd et al. 2012b). Strikingly, all of the vaccinated macaques controlled viral replication during acute infection, and 6 of 8 became elite controllers. The vaccinees demonstrated early robust CTL responses to Vif RL9 and Nef RL10 when compared to the unvaccinated macaques. CTL responses against Vif RL8 were present but equal between the two groups, suggesting that Vif RL8-specific CTLs may not differentiate elite controllers from progressors. In vaccinees that progressed, viral sequencing revealed the presence of escape mutations in all three targeted epitopes. A set of follow-up experiments that used a monotypic Nef RL10 vaccination demonstrated that Nef RL10-specific CTLs, alone, are insufficient to induce elite control of SIVmac239 (Martins et al. 2015). These data, taken together, suggest that vaccine-induced Vif-specific T-cells are key for mediating control of SIV in the context of Mamu-B*08.

These results raise the question as to whether vaccine-induced viral control will be as effective in macaques whose MHC alleles do not dominantly present Vif epitopes. The Matano laboratory shed light on this question in a recent experiment studying the vaccination of macaques with an MHC-I haplotype associated with dominant Nef-specific CD8+ T-cell responses (Iwamoto et al. 2014). They used a DNA prime/SeV-VifNef boost vaccination to determine whether Vif- or Nef-specific CTLs could be induced to control SIV infection. Indeed, they found that the frequency of Vif-specific CD8+ T-cells in the acute phase of infection was significantly higher in vaccinated controllers. In unvaccinated and vaccinated non-controller macaques, however, Nef-specific CD8+ T-cells were predominant and led to early viral escape from these T-cells. Interestingly, most of the unvaccinated and vaccinated macaques showed Nef-specific CD8+ T-cell responses in the chronic phase. This is consistent with data from our laboratory that showed a correlation between high frequencies of Vif-specific T-cell responses and low viral set points during the chronic phase (Martins et al. 2010).

## Vif-specific T-cells are correlates of decreased risk of HIV acquisition

**Fig. 2:**
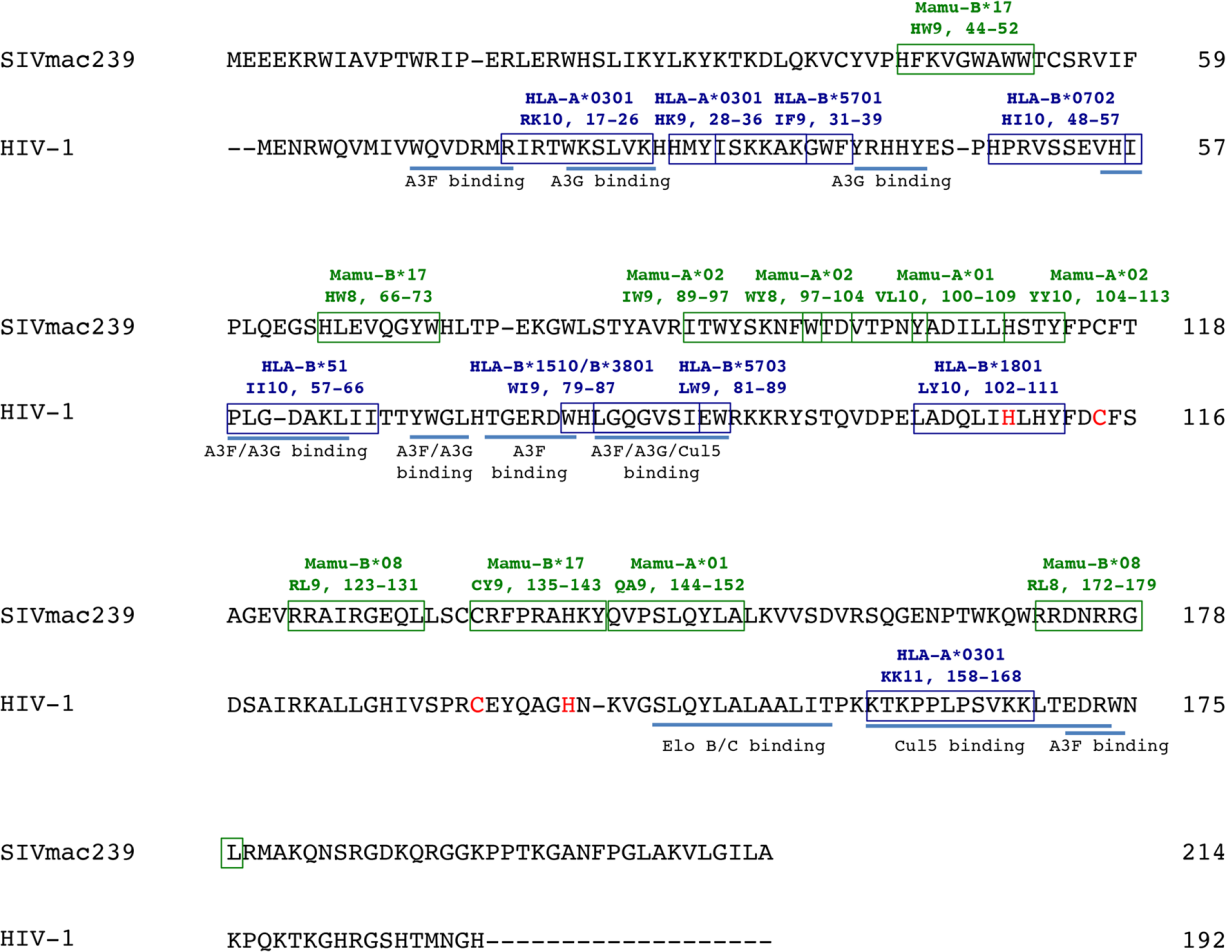
HIV and SIV Vif are targeted for presentation by MHC-I gene products. The major function of HIV/SIV Vif is to prevent incorporation of A3G into the capsid of progeny virus by targeting the Vif/A3G complex to the proteasome. In so doing, the AIDS virus is able to prevent hypermutation of its progeny virus upon infection of a subsequent cell. This Vif amino acid alignment depicts the SIV and HIV Vif minimal optimal epitopes and the MHC-I gene products that present them. In addition, we show the extent of overlap between the Vif minimal optimal epitopes and known functional domains of Vif. The HIV Vif HCCH motif (amino acids shown in *red*) binds to Cul5. References: SIVmac239 epitopes (see Table 1), HIV-1 epitopes (Llano et al. 2013), Elo B/C binding (Stanley et al. 2008), Cul5 binding (Dang et al. 2010; Luo et al. 2005; Stanley et al. 2008), A3G binding (Chen et al. 2009; Dang et al. 2009; Russell and Pathak 2007), A3F binding (Dang et al. 2010; He et al. 2008; Russell and Pathak 2007), A3G and A3F binding (Dang et al. 2010; He et al. 2008; Pery et al. 2009)

The association between elite control and certain MHC-I alleles, including the HLA-B*27 and -B*57 alleles, is widely accepted and has been reviewed extensively (Goulder and Watkins 2008). Of note, however, is that the HLA-B*27 allele shares a similar peptide binding motif as the rhesus macaque Mamu-B*08 allele (Loffredo et al. 2009). This suggests that the immunodominant epitopes, namely Vif and Nef, associated with spontaneous and vaccine-induced control of SIV may also be important in human control of HIV. Indeed, a number of HIV Vif epitopes have been shown to bind MHC-I proteins and activate CTLs (Fig. 2).

Analyses of individuals who are at high risk for infection but remain seronegative provide fertile ground for understanding protective mechanisms (Addo et al. 2011; Guerini et al. 2011). These “exposed, seronegative individuals” (ESNs) do not acquire HIV infection despite repeated exposure and their existence has been widely reported. A study conducted by Kebba et al. in 2004 suggests that ESNs target different epitopes from individuals who are infected, and that these epitopes are critical for viral replication (Kebba et al. 2004). In their study, they demonstrated that ESNs mounted high-frequency CTL responses against Vif, while seropositive individuals mounted lower frequency, subdominant responses.

A more recent study conducted by the Kallas Laboratory (part of the iPrEx chemoprophylaxis trial) examined the relationship between HIV-1-specific T-cell responses in ESNs and risk of infection (Kuebler et al. 2015). They compared the T-cell responses of ESNs who remained seronegative with those who ultimately acquired infection. Interestingly, the frequency of Vif-specific responses correlated with a substantial reduction in risk of HIV-1 infection. Their data show that a 10-fold increase in Vif-specific T-cell responses, as measured by ELISpot, corresponded to a 64% decreased risk of HIV-1 infection. Further analyses of these types of human cohorts will be required to understand the role vaccine-induced Vif-specific CTL responses could have in preventing infection.

## Conclusion

Sterilizing immunity to HIV-1 has proven difficult to achieve through vaccination (Fuchs and Desrosiers 2016). A successful vaccine should therefore aim to restrict HIV replication in the acute phase by inducing potent effector memory CD8+ T-cell responses at the portals of viral entry. There is evidence that such a T-cell response can limit the peak of acute phase infection, thereby decreasing the number of host cells that serve as viral reservoirs. A decrease in the number of viral reservoirs is believed to slow disease progression and decrease the host’s potential for transmitting the virus. Importantly, a decrease in the number of HIV-infected cells also diminishes the viral replication required for a virus to mutate and escape CD8+ T-cell pressure.

It is important to note that the Merck vaccine trials, which ended in failure, did not include a Vif immunogen (Sekaly 2008). Since these trials were designed, Vif-specific T-cell responses have been found in elite controllers and correlated with reduced viral loads during the chronic phase of infection. Evidence from human cohorts has also suggested the presence of broad Vif-specific responses in HIV-1 patients and high-risk seronegative individuals (Kuebler et al. 2015; Tarosso et al. 2010). Given the data suggesting an important role for Vif-specific T-cell responses, it will be valuable to continue studying Vif-mediated immunity and the potential role of these T-cells in both prevention and control of HIV-1 infection.

Immunodominance of viral epitopes varies for different MHC-I molecules, and recent studies have described the importance of CD8+ T-cell responses in controlling HIV/SIV viremia. Mapping of the SIV epitopes that are bound by different MHC-I molecules, especially those associated with elite control, has been an important step toward understanding control of SIV infection. We hope that this review has demonstrated how an in-depth understanding of the MHC-I gene products associated with elite control of HIV and SIV, and the epitopes that they present, can provide researchers with a glimpse into the protective immune responses that underlie AIDS nonprogression.
